# Dielectrophoretic separation of blood cells

**DOI:** 10.1007/s10544-022-00623-1

**Published:** 2022-08-25

**Authors:** Maria E. P. Emmerich, Anne-Sophie Sinnigen, Peter Neubauer, Mario Birkholz

**Affiliations:** 1 Chair of Bioprocess Engineering, Institute of Biotechnology, TU Berlin, Ackerstrasse 76, ACK24, D-13355 Berlin, Germany; 2 IHP – Leibniz-Institut für innovative Mikroelektronik, Im Technologiepark 25, 15236 Frankfurt (Oder), Germany

**Keywords:** Bioelectronics, Dielectrophoresis, Blood cells, Cell separation, Microfluidics, Lab-on-chip systems

## Abstract

Microfluidic dielectrophoretic (DEP) devices enable the label-free separation and isolation of cells based on differences in their electrophysiological properties. The technique can serve as a tool in clinical diagnostics and medical research as it facilitates the analysis of patient-specific blood composition and the detection and isolation of pathogenic cells like circulating tumor cells or malaria-infected erythrocytes. This review compares different microfluidic DEP devices to separate platelets, erythrocytes and leukocytes including their cellular subclasses. An overview and experimental setups of different microfluidic DEP devices for the separation, trapping and isolation or purification of blood cells are detailed with respect to their technical design, electrode configuration, sample preparation, applied voltage and frequency and created DEP field based and related to the separation efficiency. The technique holds the promise that results can quickly be attained in clinical and ambulant settings. In particular, point-of-care-testing scenarios are favored by the extensive miniaturization, which would be enabled by microelectronical integration of DEP devices.

## Introduction

Details of the human blood composition have an immense medical potential as they can be used as a diagnostic tool to provide information about the health status of a patient, diseases and their prognosis. To date the analysis of a whole blood sample requires the utilization of comparatively large devices and skilled personal in laboratories, which is time and cost intensive. Microfluidic dielectrophoretic (DEP) devices in the format of “Lab-on-a-Chip” (LoC) systems might represent a suitable alternative for the analysis of blood cell composition in future diagnostics or for the label-free separation of cells in medical research, diagnostics and therapy (Gambari et al. 2007).

Remarkably, DEP is a purely electrical effect and offers the perspective for miniaturization and point-of-care testing (POCT) when combined with modern techniques of microelectronics (Birkholz et al. 2016) POCT devices can be used for various applications including the detection of diseases (Jung et al. 2015). They have to be able to produce fast results that should be comparable to the devices conventionally used in laboratories for analysis and should be easy to handle in order to be utilized by non-trained staff (Jung et al. 2015).

Previous reviews described the biomedical potential of DEP including the separation of bacterial cells (Pitt et al. 2016) or DNA (Das and Kelley 2020), rare cells (Borgatti et al. 2008; Hyun and Jung 2013; Jubery et al. 2014; Perez‐Gonzalez et al. 2016; Iliescu et al. 2019), its potential in regard to the detection of malaria (Aditya et al. 2013; Kasetsirikul et al. 2016) or brief descriptions of applications using the DEP effect in respect to blood or blood cells (Kumar et al. 2019; Pethig et al. 2010; Sarno et al. 2021). Additionally, the separation of blood cells was detailed to some extent (Demircan et al. 2013; Jubery et al. 2014; Perez‐Gonzalez et al. 2016; Waheed et al. 2021). Since 2000 this output has increased, signifying the growing interest in the field. However, to date there is no comprehensive review on the dielectric separation of blood cell subtypes especially including the separation of leukocyte subtypes.

On average an adult human male has about 5 L of blood that is composed of ca. 3 L plasma and ca. 2 L cells. The latter consists of erythrocytes (red blood cells, RBCs), thrombocytes (platelets, PLTs) and leukocytes (white blood cells, WBCs), with 3 subclasses [lymphocytes (T cells and B cells), monocytes and granulocytes]. Granulocytes are divided into eosinophils, basophils and neutrophils (Fischbach and Dunning 2009).

The complete blood count (CBC) distinguishes between the different blood cell subtypes and specifies their concentrations in number per mm^3^ or µL (Brugnara and Kratz 2015). The depletion or increase of a specific cell count could indicate, among other symptoms, the presence of an acute infection, or a genetic disease, as well as the health-related quality of life or an increased mortality risk (Jung et al. 2015; Wouters et al. 2021). Determining the distribution of leukocyte subtypes has many applications in medical research and diagnostics, as it is closely linked to the status of the immune system (Murphy et al. 2018). Abnormalities in the number of leukocyte subtypes can be related to infections and diseases such as inflammations, coronary heart diseases and cancer (Wang et al. 2018). For example, stress trauma and infection as well as pregnancy or some common medications lead to an increased leukocyte count (leukocytosis). Basophilia and eosinophilia could indicate an allergic reaction, monocytosis a viral infection, lymphocytosis and eosinophilia a hypersensitivity reaction and neutrophilia a chronic inflammation (Mank and Brown 2021; Riley and Rupert 2015), to mention only a few examples. Cells present in blood, their concentrations and examples of related diseases are compiled in Table 1. As-normal defined concentrations of the individual cell type can vary as they depend on factors like age, gender and ethnic background (Fischbach and Dunning 2009; Wouters et al. 2021). Values shown in this table are given for a white, adult, male human (Bain and Huhn 1997).

**Table 1:** Concentrations of blood cells and possibly connected disease (Fischbach and Dunning 2009), percentages of WBC from (Estridge and Reynolds 2012)

Cell type	Normal conc. in blood [cells µL^−1^]	Percentage of total WBC [%]	Function	Connected Diseases	Abnormal conc. in Blood [cells µL^−1^]
Erythrocytes	4.2–5.4 × 10^6^	/	Oxygen Transport	Anaemia (e.g. in Hodkin´s Disease, Addison´s Disease) Erythrocytosis (e.g. in Polycythemia vera)	Depending on the type of anaemia, decreasing number
Thrombocytes	1.4–4 × 10^5^	/	blood clotting vascular integrity vasoconstruction	Thrombocytosis (e.g. in rheumatoid arthritis, Splenectomy, Ashyxiation)	< 10^6^ < 4 × 10^5^ (Wouters et al. 2021)
				Thrombocytopenia (e.g. in viral/bacterial infections, HIV infections, ethanol abuse)	> 0.5 × 10^5^
Lymphocytes	1.5–4.0 × 10^3^	25–40	response against viral and bacterial infections antibody production (B cells)	Lymphocytosis (e.g. in acute HIV infection, Pneumonia, acute viral Hepatitis)	> 4 × 10^3^
				Lymphopenia (e.g. in Hodkin´s Disease, Aplastic anemia)	< 1 × 10^3^
Monocytes (Monomorpho-nuclear)	0.1–0.5 × 10^3^	3–9	phagocytosis removal of injured or dead cells response to “severe” infections	Monocytosis (e.g. Syphilis, Tuberculosis)	> 0.5 × 10^3^
				Decreased Monocyte Count (e.g HIV, aplastic Anemia)	< 0.1 × 10^3^
Basophils	0.02–0.05 × 10^3^	0–1	contain messengers as histamine and heparin in granules of the cell response to parasitic infections response to some allergic diseases	Basophilia (e.g. Hodkins Disease, Granulocytic-/ Acute basophilic Leukemia)	> 0.05 × 10^3^ > 0.2 × 10^3^
				Basopenia (e.g. in acute phase of infection, Stress reactions)	< 0.02 × 10^3^
Eosinophils	0–0.7 × 10^3^	1–3	Contain messengers as histamine and heparin in granules of the cell reaction to later stages in inflammation response to allergic diseases and parasitic infections	Eosinophilia (e.g. in allergies, asthma, parasitic diseases)	> 500 × 10^3^
				Eosinopenia (e.g. in Cushing´s syndrome, acute bacterial infections)	< 0.05 × 10^3^
Neutrophils	3–7 × 10^3^	50–72	reaction to inflammation phagocytosis primary defence against microbial infections and cancer	Neutrophilia (e.g. in bacterial infections, chronic and acute inflammations, some viral infections, sepsis)	> 8.0 × 10^3^
				Neutropenia (e.g. in Hepatitis, Malaria, Influenza)	< 1.8 × 10^3^

Blood counts are currently conducted with the help of bench-top hematology analyzers which are based on either light scattering (flow cytometry) or resistive pulse sensing (Coulter principle). These devices usually do not require sample preparation or skilled personnel and are able to deliver results within a few minutes. However, they are of comparably high cost, space consuming and are available in specific laboratories only (Brugnara and Kratz 2015; Jung et al. 2015).

State-of-the-art blood cell analyses are usually performed by methods like fluorescence-assisted cell sorting FACS, magnetic activated cell sorting MACS and flow cytometry (Yang et al. 2000) Sample preparation for these types of cell separations is time-consuming. In contrast, a dielectrophoretic isolation of cells would not need any staining of other pretreatment procedure. Therefore, the isolated cells would remain in a natural state and thus more reliable results would be likely obtained in subsequent analyses (Yang et al. 2000).

## Dielectric properties of blood cells

The dielectrophoresis effect is observed for polarizable particles within a non-uniform, oscillating electric field *E* causing the induction of a dipole moment *p*_in_ in the particle. The resulting dielectrophoretic force *F*_DEP_ = *p*_in_*∇E* scales with three factors (Pohl 1951): (i) 2π*r*^3^ $\varepsilon$_m_, which corresponds to the product of the volume of a spherical particle and the dielectric constant $\varepsilon$_m_ of the medium, (ii) the real part of the Clausius Mossotti factor *Re*(*K*_CM_), which is a function of the complex dielectric constants $\varepsilon$^*^of medium and cell *K*_CM_*(*$\varepsilon$_m_*, $\varepsilon$_c_*), as well as (iii) the vector gradient of the square of the electrical field *∇ E*^2^.

Solving *∇ E*^2^ by applying the chain rule yields *2 E* *∇ E*, which according to electrostatic multipole development describes the interaction between electrical field gradient and induced dipole moment (Birkholz 1995). In contrast to electrophoresis, the DEP effect can thus occur for uncharged particles as well. In biological cells the induced dipole develops due to the spatial redistribution of electrical charges located on the surface of the cell membrane both in intra- and extracellular space (Pohl 1951). The Clausius–Mossotti factor *K*_CM_ is given by

1 $${K}_{\mathrm{CM}}=\frac{{\varepsilon }_{\mathrm{c}}^{*}-{\varepsilon }_{\mathrm{m}}^{*}}{{\varepsilon }_{\mathrm{c}}^{*}+2{\varepsilon }_{\mathrm{m}}^{*}}$$

with materials constants depending via $\varepsilon$* = $\varepsilon$ – iσ*/(2πF)* on the real part $\varepsilon$ of the complex dielectric constant ℇ*, the frequency *f* of voltage oscillations and electrical conductivities $\sigma$_c_ and $\sigma$_m_ of cell and medium, respectively (Pethig 2017a). For reasons of brevity, the dielectric constant of a specific material $\varepsilon$_x_ = $\varepsilon$_0_ $\varepsilon$_x,r_ is understood throughout this review as the product of vacuum permittivity $\varepsilon$_0_ and a dimensionless constant $\varepsilon$_x,r_. Expressing Re(*K*_CM_) by its real and complex components reveals its dependence on the applied frequency and the electrical properties of cells and the suspending medium,

2 $$Re\left({K}_{\mathrm{CM}}\right)=\frac{{\omega }^{2}\left({\varepsilon }_{\mathrm{c}}-{\varepsilon }_{\mathrm{m}}\right)\left({\varepsilon }_{\mathrm{c}}+2{\varepsilon }_{\mathrm{m}}\right)+\left({\sigma }_{\mathrm{c}}+2{\sigma }_{\mathrm{m}}\right)\left({\sigma }_{\mathrm{c}}-{\sigma }_{\mathrm{m}}\right)}{{{\omega }^{2}({\varepsilon }_{\mathrm{c}}+2{\varepsilon }_{\mathrm{m}})}^{2}+2{({\sigma }_{\mathrm{c}}+2{\sigma }_{\mathrm{m}})}^{2}}$$

The real part of the Clausius–Mossotti factor is a dimensionless quantity and ranges from -0.5 to 1. Its sign determines whether the blood cell will experience an attraction (positive DEP effect) or a repulsion (negative DEP effect) towards field regions with larger *∇ E*^2^ values. The switching of signs occurs at the so-called crossover frequency *f*_co_, and its value can be derived by setting Re(*K*_CM_) equal to zero resulting in.

3 $${f}_{c\mathrm{o}}^{2}=\frac{1}{4{\pi }^{2}}\frac{\left({\sigma }_{\mathrm{m}}-{\sigma }_{\mathrm{c}}\right)\left({\sigma }_{\mathrm{c}}+2{\sigma }_{\mathrm{m}}\right)}{\left({\varepsilon }_{\mathrm{c}}-{\varepsilon }_{\mathrm{m}}\right)\left({\varepsilon }_{\mathrm{c}}+2{\varepsilon }_{\mathrm{m}}\right)}$$

At this oscillation frequency, the assumingly homogeneous cell will remain unaffected by any DEP effect (Labeed and Fatoyinbo 2015), albeit this only applies to viable cells with undamaged cell membranes (Pethig 2010).

The dielectric properties of biological cells are more complex than those of a homogeneous particle. To model their internal structure, with their membrane and cytoplasm, the calculations would have to be solved individually for these structures (Gascoyne et al. 1995). This can be described by the single-shell model, which simply assumes the cytoplasm as a sphere of radius *r* and the cell membrane as a surrounding shell of thickness *d* (Pethig 2017b). The resulting complex permittivity *ε*_c_* of the cell is calculated by

4 $${\varepsilon }_{\mathrm{c}}^{*}={\varepsilon }_{\mathrm{mem}}^{*}\frac{{\left(\frac{r+d}{r}\right)}^{3}+2\left(\frac{{\varepsilon }_{\mathrm{int}}^{*}-{\varepsilon }_{\mathrm{mem}}^{*}}{{\varepsilon }_{\mathrm{int}}^{*}+2{\varepsilon }_{\mathrm{mem}}^{*}}\right)}{{\left(\frac{r+d}{r}\right)}^{3}-\left(\frac{{\varepsilon }_{\mathrm{int}}^{*}-{\varepsilon }_{\mathrm{mem}}^{*}}{{\varepsilon }_{\mathrm{int}}^{*}+2{\varepsilon }_{\mathrm{mem}}^{*}}\right)}$$

where subscript *int* stands for the cell’s interior, while *mem* indicates the membrane (Gascoyne et al. 1995). The approach can be extended to a multi-shell model to include more details like the nucleus or cell organelles (Cottet et al. 2019). Even though the single-shell model averages over the whole cytoplasm, which is in fact heterogeneously structured, it allows a quite accurate calculation of *Re*(*K*_CM_) and therefore an estimation of the possible cell trajectory within an inhomogeneous electrical field (Turcu and Lucaciu 1989).

The charge cloud surrounding the cell is typically positively charged, whereas a negative charge accumulates on its inside of the cell membrane. Due to the separation of charges across the plasma membrane, it is acting as an electrical capacitor with a thickness *d* of about 6—10 nm (Pethig 2017b). The capacitance of the plasma membrane depends on its composition and characteristic properties like hydrophobicity (Golowasch and Nadim 2014; Pethig 2017b), which is closely linked to cell function and thus differs among various cell types. For biological cells the radii are much larger than the thickness of the membrane, *r* > > *d*, this allows a simplified calculation of the specific capacitance by *c*_mem_ = *C*_mem_/*A* = ℇ_mem_/*d* (Pethig and Kell 1987). The area of the membrane *A* is a major parameter for the determination of dielectrophoretic properties of cells (Gascoyne et al. 2013). Morphological features like microvilli and folds of the cell surface are also related to the dielectric properties of the cells, since they cause a modification of the surface area (Wang et al. 1994; Yang et al. 1999). Furthermore, changes in the capacitance values can indicate changes within the cell like e.g. death or fertilization (Blinks 1930; Cole 1937; Pethig 2017b).

The introduction of the specific capacitance *c*_mem_ allows to reformulate the crossover frequency *f*_co_, according to.

5 $${f}_{c\mathrm{o}}\approx \frac{{\sigma }_{\mathrm{m}}}{\sqrt{2}\pi r{c}_{\mathrm{mem}}}$$

This formula shows the dependence of the crossover frequency *f*_co_ on the conductivity of the medium and that it is inversely proportional to the cell radius and the capacitance of the membrane (Chan et al. 1997; Gascoyne et al. 1997). It has been developed within the framework of the single shell model and holds true for experimental set-ups, where the conductivity of the shell, i.e. the plasma membrane, is much lower than that of the surrounding medium, σ_mem_ < < σ_m_; an assumption that is typically obeyed.

Dielectric properties of human blood cells have been investigated in various studies and a set of determined values for permittivity of cell membrane ɛ_mem_ as well as cytoplasm ɛ_int_ is presented in Table 2 (Aghaamoo et al. 2019; Becker et al. 1995; Gascoyne et al. 2004; Nada et al. 2018; Yang et al. 1999). These published values show some scatter. In addition to those listed, various studies that use other mammalian blood cells are not included. Occasionally, $\varepsilon$ values for the cytosol of a cell that exceed the dielectric constant of water, $\varepsilon$ > $\varepsilon$_H2O_ = 80$\varepsilon$_0_, appear in the literature. These values have often been shown to shift back into the expected range ε < ε_H2O_ when modeling the entire cell with a sufficiently complex model (Pethig 2010). In the case of lymphocytes, for example, a single-shell model is not sufficient. Rather, a double-shell or a three-shell model must be used, where the nucleus is modeled as a particle in the cytosol, to correctly calculate the dielectric behavior of lymphocyte suspensions (Asami et al. 1989; Cottet et al. 2019). We have compiled in Table 2 what seem to us to be the best values from the literature within the framework of their corresponding models. The experimental methods used to determine these parameters are often either electrorotation (ROT), or dielectric spectroscopy. For these measurements, the cells are exposed to various frequencies and the results are interpreted using parameter fitting, in order to determine their dielectric properties (Markx and Davey 1999).

**Table 2:** Dielectric properties of human blood cells

Cell type	Radius [µm]	σ_cyto_ [S m^−1^]	ε_cyto_	Membrane thickness [nm]	C_mem_ [mF m^−2^]	σ_mem_ [mS m^−1^]	ε_mem_	Method	Ref.
Erythrocytes	3.3, axial ratio 1:2	0.4	212	8	9.97			ROT, to 15 MHz	(Gimsa et al. 1996)
	3.3, axial ratio 1:2	0.535	50	8	9.97			ROT, from 15 MHz	(Gimsa et al. 1996)
	2.4	0.53	50	8		10^-3^	9.04	Impedance, dielectrophoresis, ROT	(Gimsa and Wachner 1998)
	3.95 ± 0.25							imaging	(Techaumnat et al. 2020)
		0.31 ± 0.03	59 ± 6	4.5		< 10^–6^	4.44 ± 0.45	DEP levitation and trapping	(Gascoyne et al. 2002)
	2.7			3.1			5 ± 0.05	Time domain dielectric spectroscopy	(Lisin et al. 1996)
	3.36 ± 0.25, axis ratio 1:2	0.535			8.2			ROT	(Gimsa et al. 1994)
	**2.8 ± 0.1**				**9 ± 0.8**			**ROT**	(Gascoyne et al. 1997)
		0.52 ± 0.051	57 ± 5.4		9 ± 0.8			ROT, 10 kHz bis 10 MHz	(Becker et al. 1995)
		0.52 ± 0.05	57 ± 5.4		12 ± 1.2			ROT	(Gascoyne et al. 1997)
Thrombocytes	1.1	0.16	50	8		10^-4^	7.2	ROT	(Egger et al. 1988)
	**0.9**	**0.25**	**50**	**8**		**10**^**-6**^	**5.5**	**ROT**	(Egger and Donath 1995)
Leukocytes									
T-Lymphocytes		0.76 ± 0.058	64 ± 5.9		11 ± 1.1			ROT	(Becker et al. 1995)
	3.6 ± 0.55	0.53 ± 0.1	100		7.01 ± 0.91			ROT	(Han et al. 2013)
	4.75 ± 0.12	0.5						ROT	(Keim et al. 2019)
	**3.29 ± 0.35**	**0.65 ± 0.15**	**103.9 ± 24.5**		**10.5 ± 3.1**			**ROT**	(Yang et al. 1999)
	3.40 ± 0.08				13.29 ± 1.82			Dielectric crossover frequency	(Vykoukal et al. 2009)
B-Lymphocytes	3.6 ± 0.6	0.41 ± 0.1			10.33 ± 1.6			ROT	(Han et al. 2013)
	**3.29 ± 0.26**	**0.73 ± 0.18**	**154.4 ± 39.9**		**12.6 ± 3.5**			**ROT**	(Yang et al. 1999)
	4.1 ± 1.4	0.55 ± 0.07			10.14 ± 0.08			ROT and imaging	(Huang et al. 2018)
	3.09 ± 0.22				9.91 ± 0.8				(Vykoukal et al. 2009)
Monocytes	4.8 ± 0.55	0.37 ± 0.15			11.77 ± 2.12			ROT	(Han et al. 2013)
	**4.63 ± 0.36**	**0.56 ± 0.10**	**126.8 ± 35.2**		**15.3 ± 4.3**			**ROT**	(Yang et al. 1999)
	4.21 ± 0.05				14.23 ± 0.81			Dielecric crossover frequency	(Vykoukal et al. 2009)
Granulocytes	4.3 ± 0.55	0.31 ± 0.06			9.14 ± 1.06			ROT	(Han et al. 2013)
	**4.71 ± 0.23**	**0.60 ± 0.13**	**150.9 ± 39.3**		**11.0 ± 3.2**			**ROT**	(Yang et al. 1999)
Neutrophils	4.06 ± 0.06				9.84 ± 0.07			Dielectric crossover frequency	(Vykoukal et al. 2009)
Eosinophils	4.19 ± 0.07				9.39 ± 0.41			Dielectric crossover frequency	(Vykoukal et al. 2009)
Basophils	3.58 ± 0.03				11.2 ± 1.25			Dielectric crossover frequency	(Vykoukal et al. 2009)

**Fig. 1:**
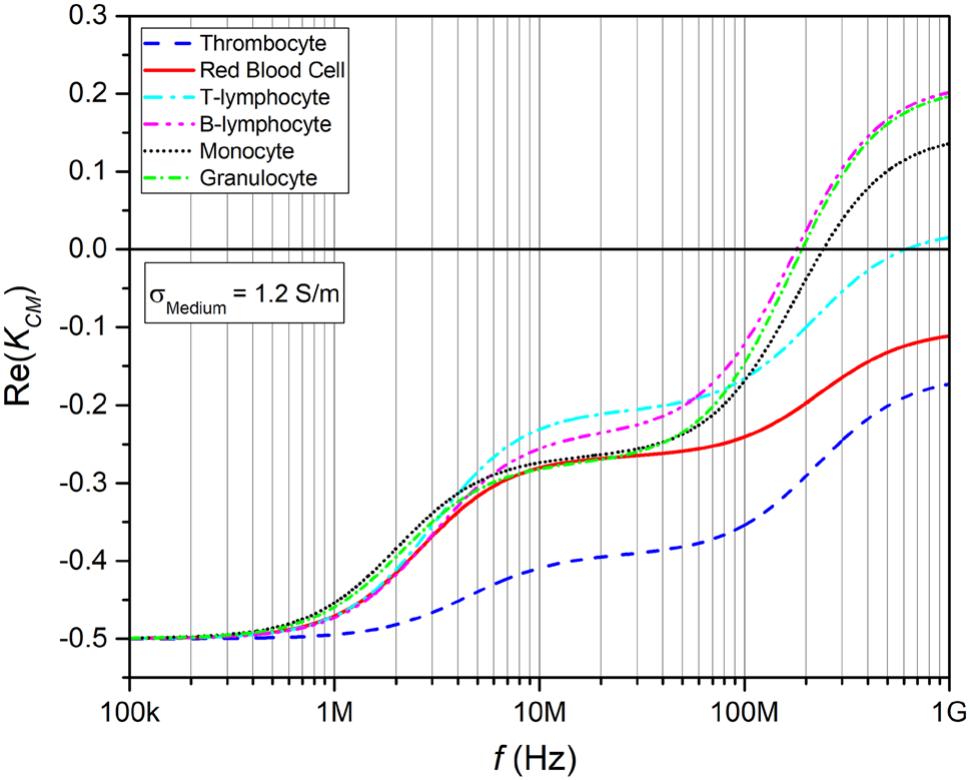
Re(K_CM_) of blood cells in blood plasma, values calculated using the MyDEP software (Cottet et al. 2019), the volume fraction is set to v = 0.45, the cells are each defined by a single shell model. The crossover frequency can be seen by the transition of negative to positive values

**Fig. 2:**
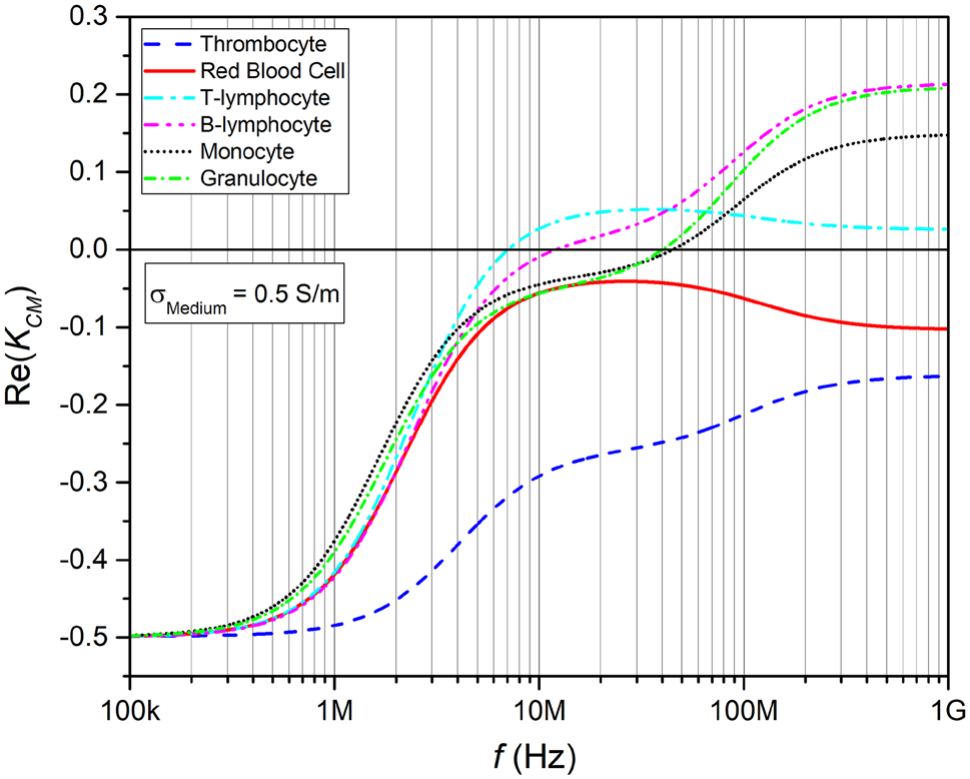
Re(K_CM_) of blood cells in a medium with σ_m_ = 0.5 S m^−1^ and ε_m_ = 80, values calculated using the MyDEP software (Cottet et al. 2019), the volume fraction is set to 0.45, the cells are each defined by a single shell model. The crossover frequency can be seen by the transition of negative to positive values

*ROT* electrorotation, values labeled as bold were chosen in myDEP database and are plotted in the following Figs. 1 and 2

The membrane capacity of the cell is another important value to evaluate its behavior within the DEP field as it determines if the cell experiences a positive or a negative DEP effect. The values of the membrane capacity of a specific cell type differ within the literature as well (Becker et al. 1995; Gascoyne et al. 2013; Gascoyne et al. 1997; Han et al. 2011; Nada et al. 2018; Piacentini et al. 2011; Vykoukal et al. 2009; Yang et al. 1999). Table 2 also shows the cell membrane capacities of the blood cells in relation to the conducted studies. Monocytes and granulocytes represent the largest cells in blood (Murphy et al. 2018).

The mean capacitance and cell size of B and T cells is similar, which makes their dielectrophoretic separation more challenging (Yang et al. 1999).

The real part of the Clausius–Mossotti factor Re(*K*_CM_) for the different blood cell types in blood plasma is shown in Fig. 1 as a function of frequency between 100 kHz and 1 GHz. The plotted values have been calculated using the free software myDEP (Cottet et al. 2019). We have labeled the values in Table 2 as bold that were chosen in the myDEP database and are plotted in the Re(*K*_CM_) plots below. For the conductivity of the medium the value *σ*_m_ = 1.2 S m^−1^ was inserted. In the low frequency range, the Re(*K*_CM_) curves start at a value of -0.5 and all cell types are repelled from the spatial regions of high electric field density, representing the case of negative dielectrophoresis. The four types of white blood cells exhibit crossover frequencies of a few 100 MHz, where they would switch to positive DEP. Thrombocytes and red blood cells, however, would show no crossover in the displayed frequency range and would only suffer negative DEP in blood plasma**.**

## Buffer and cell concentration

The cells’ electrophysiological parameters ε and σ have a strong effect on the DEP force as they are influencing the real part of the Clausius–Mossotti factor *Re(K*_CM_*)*, see Eqs. (1) and (2). However, the suspending medium or buffer may affect the strength of the DEP force *F*_DEP_ used for cell separation as well and should be chosen in dependence of the experiment type. The experiments analyzed in this review used a variety of buffers and cell concentrations that are compiled in Table 3.

**Table 3:** Buffer Conductivity and total cell concentration used in the experiments reviewed

Buffer used	Conductivity [S m^−1^]	Frequency [Hz]	Cell density [ml^−1^]	Applied Voltage [V_pp_]	Ref.
Whole Blood (anticoagulated with Heparin-Agarose) diluted 1:5 with PBS	1.7	2 M	1 × 10^6^*	3	(Han and Frazier 2008)
Low Conductivity Suspension Medium with Inostol	0.17	100 k	RBC N/A	5.8	(Han et al. 2011)
			5 × 10^6^ WBC		
Suspension Buffer	1	200 k	1 × 10^7^ malignant	5	(Wang et al. 1995)
8.5% w/v sucrose, 0.3% w/v dextrose with hemidodium EDTA			3 × 10^7^ blood cells		
PBS diluted in sucrose solution (PBS, sucrose, 1% BSA, EDTA)	0.055	100 k	1–2 × 10^8^	10	(Piacentini et al. 2011)
Standard Tyrode´s buffer (10 mM HEPES, 2 mM MgCl_2_, 137.5 mM NaCl, 12 mM KCl, 5 mM glucose, 0.1% BSA)	1.7	1 M	10^7^ platelets	100	(Pommer et al. 2008)
			N/A non-platelets		
Low Electric Conductivity Buffer (LEC buffer)	0.05	1 M	10^7^ platelets	100	(Pommer et al. 2008)
			N/A non-platelets		
8.5% sucrose/0.3% dextrose (wt/wt) buffer	0.01	10 – 40 k	2 × 10^6^	4	(Yang et al. 2000)

^*^assuming typical blood concentrations as shown in Table 1

*f*_co_ to lower frequencies, the conductivity of the surrounding medium should be comparatively low. For a conductivity of σ_m_ = 0.5 S m^−1^, for instance, *f*_co_ values of leucocytes would shift to one to two magnitudes lower frequencies compared to plasma medium as shown in Fig. 2

The conductivity of a saline solution with a density of 0.1% (w/v) is about 0.2 S m^−1^ (Culkin 1986) whereas the conductivity of blood plasma varies in literature and depends on its components, especially its glucose levels (Topsakal et al. 2011); its value is documented between 1.2 and 1.8 S m^−1^ (Ahmad and Rauf 2013; Hirsch et al. 1950; Labeed and Fatoyinbo 2015; Liao et al. 2013; Topsakal et al. 2011). The crossover frequency *f*_co_ is related to the conductivity of the surrounding medium. In order to shift.

According to Eq. (5), the crossover frequency *f*_co_ of blood cells is also closely linked to their membrane capacitance. The effect was investigated by Gascoyne et al*.* using an aqueous solution with a conductivity of 30 mS m^−1^ as a medium and the results are depicted in Table 4. In these experiments the values differ between cell types, which is useful when only one specific cell type should not be impacted by the DEP force at all (Gascoyne et al. 2013). The values for red blood cells are higher than for the other cells (Gascoyne et al. 2013), followed by B cells and T cells, the three smallest cells of the listed cell types.

**Table 4:** Crossover frequency of blood cells

Cell Type	*f*_*CO*_ [kHz]^a^
Erythrocytes	268 ± 23.8
	218 ± 21.8
T cells	149 ± 20.2
	176 ± 17.6
	184 ± 22.8
B cells	163 ± 45.3
	221 ± 17.8
Monocytes	95 ± 26.8
	113 ± 6.4
Granulocytes	130 ± 37,9
	95 ± 13.9
Neutrophils	170 ± 1.2
Eosinophils	172 ± 7.3
Basophils	169 ± 18.8

^a^of the respective experiment, described above (Gascoyne et al. 2013)

The medium may have an influence on the cell morphology as well. Cells suspended in a hypertonic medium absorb water from the surrounding media which would cause an enlargement of the cell and therefore also an enlargement of the cell membrane. This results in a stretched and therefore flattened membrane which might reduce the number of ruffles. Since the shape of the cell membrane impacts its specific capacitance *c*_m_, this may result in a lower capacitance of the cell membrane (Pethig 2017b). The effect may also explain the differing dielectric values as obtained in different studies, see Table 4. Additionally, to ensure the viability of the investigated cells the medium conductivity is comparatively high. These higher values might result in an electrode polarization, especially at sub-MHz frequencies. This means that a large impedance forms at the electrode/electrolyte interface due to the charge accumulation at the electrode surface. Subsequently, the electric potential would drop and the dielectrophoresis effect would decrease. This adverse effect can be reduced by increasing the electrode surface by employing nanostructured electrodes (Koklu et al. 2017, 2016).

Finally, it should be pointed to the fact that an increase of medium conductivity would also lead to an increased power dissipation of the applied voltage in form of Joule heating and an increase in temperature (Voldman 2006). An increase of the temperature of more than 4°C over the physiological temperature can lead to cell death and should be avoided (del Rosal et al. 2013; Voldman 2006; Yan and Wu 2008).

When using blood samples one of the salts of EDTA is typically included to prevent blood clotting. This also reduces blood clotting in a microchannel. K_2_EDTA in a final concentration of 1.5 to 2.2 mg ml^−1^ is recommended and should not be increased as too high concentrations have negative effects on cell morphology (Bain 2015). Should the blood sample coagulate in a microchannel, the channel walls can be coated with bovine serum albumin to reduce interactions of cells which also reduces cell adhesion (Techaumnat et al. 2020).

## Schemes for the dielectrophoretic manipulation and separation of blood cells

Several electrode, insulator or microfluidic designs of DEP lab-on-a-chip systems can be distinguished. These include reservoir-based DEP which helps to focus, trap and sort particles by exploiting the characteristic ***E*** field gradients at the junctions of the reservoir to the microchannel (Kale et al. 2014), optically-induced DEP which utilizes a photoconductor to create an optically induced ***E*** field (Zhu et al. 2010), contactless DEP where insulating microbarriers separate electrodes and samples (Li et al. 2013a), insulator-based DEP (Aghilinejad et al. 2017; Crowther and Hayes 2017), its sub type curvature-induced DEP in which the microchannel turns shape the ***E*** field (Zhu and Xuan 2011) and electrode-based DEP (Han and Frazier 2008; Han et al. 2011; Piacentini et al. 2011; Pommer et al. 2008; Yang et al. 2000) using 2D or 3D electrodes. The electrode based group of LoCs makes use of electrodes or electrode arrays that are integrated in the microchannel (Demierre et al. 2007). In contrast, insulator-based DEP, also called electrodeless DEP, is defined by channel insulators and conductive media. Problems like electrolysis, fouling or bubble formation can be avoided using insulator-based DEP, but as they apply a voltage of a few V only, they do not allow the formation of high field gradients (Crowther and Hayes 2017; Demierre et al. 2007). Advantages and disadvantages of these various designs to induce a ***E*** field gradient are listed in Table 5.

**Table 5:** Advantages and Disadvantages of DEP geometries/types

Type of DEP	Advantages	Disadvantages	Ref.
Electrode-based	High ***E*** field gradient	Electrode fouling, complex fabrication	(Kale et al. 2018; Pethig 2017c)
3D electrode based	Higher throughput	Complex fabrication, electrode fouling,	(Li et al. 2013a; Techaumnat et al. 2020; Zeinali et al. 2015)
Insulator-based	Facilitated fabrication	Required high voltages lead to joule heating	(Kale et al. 2018; Li et al. 2013a)
Reservoir-based	Heat sink reduces heating	Limited ∇***E***^2^ strength	(Kale et al. 2018, 2014)
Curvature-induced	No high-intensity local ***E*** fields	Electrode fouling, complex fabrication and operation	(Kale et al. 2018; Li et al. 2013a)
Contactless	No sample contamination, no bubbles	Complex fabrication	(Li et al. 2013a)
Optically-induced	Dynamic electrodes	Reduced cell viability	(Chu et al. 2020; Liang et al. 2014)

However, the following section is focused on electrode and insulator-based DEP as these are the most common DEP designs when working with blood cells. This holds especially for electrode based DEP for the selective isolation of biological cells whereas insulator based DEP is useful for selection based on surface charge differences (Pethig 2017c). Joule heating, as mentioned above, caused by the required higher electrical potential is a prominent problem as the temperature increase can impact cell viability (Aghilinejad et al. 2017; Voldman 2006).

In DEP systems, important factors that impact cell viability are electroporation and -lysis. When applying a strong electrical field the imposed transmembrane potential leads first to reversible then irreversible nanoscale pores in the phospholipid bilayer of the cell membrane (Bao et al. 2011). This scales with the applied electric field and cell radius in relation to the applied frequency and a time constant *τ*. The latter describes the ratio of the membrane specific capacitance *c*_mem_ and conductance σ_mem_ in relation to the cytoplasmic and medium resistivities (Voldman 2006). It is necessary to consider both the applied amplitude *V*_rms_ and frequency *f* in relation to the conductivity of the medium σ_m_ and electrode geometry in order to assure cellular intactness.

**Fig. 3:**
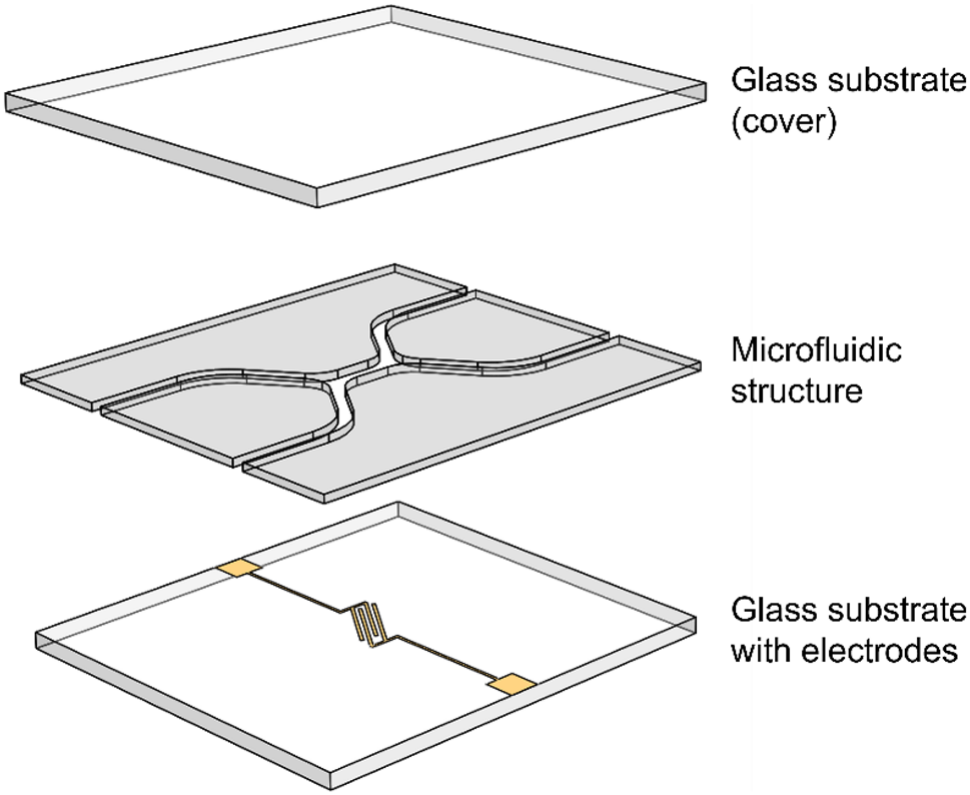
Generalized exploded-view drawing of a DEP microfluidic flow cell with bottom electrode design. The system is made up of two glass plates in between the electrodes and the polymer layer including the microfluidic channels are positioned

The main forces acting on a particle in the following set ups are the DEP force ***F***_DEP_ and the force of the flow ***F***_flow_. The sedimentation or gravitational force ***F***_grav_ can often be neglected when using blood cells due to their small size, although it has to be considered in the so-called field-flow fractionation technique, see below. The interplay of the different forces can meanwhile be simulated quite well using techniques that make use of finite element methods (FEM). In this way, the trajectories of each cell in the microfluidic channel can be followed, as also other effects such like the ∇(***E***^2^) term in the channel scaling with the DEP force (Barai et al. 2019) or the impedance matching necessary in the electronic circuit to actually bring the full voltage until to the DEP electrodes (Frey et al. 2021). The most common microfluidic LoC devices are based on a bottom electrode-based design, as it allows a facilitated fabrication of the device compared to more complex electrode geometries (Abt et al. 2020). The architecture of each device is often similar; a general scheme is depicted in Fig. 3. The microfluidic flow cell is made up of a glass substrate on top of which metallic electrodes were deposited. Above the latter, the microfluidic channel is positioned often made from PDMS (Han and Frazier 2008), photoresist resins like SU-8 (Piacentini et al. 2011), polyimide (Pommer et al. 2008), Teflon (Yang et al. 2000), UV curing epoxy glue (Gascoyne et al. 1997), dry film laminates or double-sided tape (Jeon et al. 2017; Liao et al. 2013).

The electrode and microfluidic geometries are usually fabricated using photolithography techniques which were adapted from the microelectronics industry (Birkholz et al. 2016; Garcia-Cordero and Ricco 2008). Yet, the microfluidic layer is made up off less traditional materials like low-cost polymeric materials instead of glass or silicon, because of their lower cost and easier manufacture (Yan and Wu 2008). The biocompatibility of dry film, SU8 and PDMS has been documented by their respective selling sites, however the hemocompatibility of glass, silicon, silicon nitride and especially SU8 has been described as low (Piacentini et al. 2011; Weber et al. 2018; Weisenberg and Mooradian 2002). On the other hand PDMS, silicon dioxide and parylene thin films have been documented to be hemocompatible (Piacentini et al. 2011; Weisenberg and Mooradian 2002). Additionally, the addition of an anticoagulant or the addition of bovine serum albumin can reduce cell material or channel interactions and therefore the deleterious effects of some materials on blood (Piacentini et al. 2011; Techaumnat et al. 2020).

It is common to use a microfluidic pump system to establish a flow inside the chamber (Wang et al. 1995; Han and Frazier 2008; Pommer et al. 2008; Yang et al. 2000). Capillary force is used as well, but less common (Liao et al. 2013). To establish a stable flow a withdrawal pump can be of use. The required alternating voltage of defined frequency *f* typically ranges between 0.05 and 50 MHz and is mainly supplied by a desk-top signal generator. However, it has recently been shown that the voltage usually set on commercial function generators rarely reaches the DEP electrodes in their entirety (Boldt et al. 2021; Frey et al. 2021). Consequently, special measures must be taken to match the impedance of the supply lines and electrode system. For evaluating the experiment, researchers use optical microscopy for following directly the cells’ trajectories (Wang et al. 1995; Han and Frazier 2008; Han et al. 2011; Liao et al. 2013; Piacentini et al. 2011), a hemacytometer (Han and Frazier 2008) or flow cytometry (Pommer et al. 2008; Yang et al. 2000, 1999).

The methods for cell separation by deflection of the cells using negative DEP can be distinguished by the electrode geometry with respect to the flow. The geometry is either based on one or more electrode beams at an inclined angle or at a 90° angle with respect to the flow direction.

The inclined electrodes could be facing towards the direction of the stream or against it (Han and Frazier 2008), and their angle influences the separation efficiency (Han et al. 2011). Moreover, it is also possible to trap the cells by using a quadrupole electrode array or in dead end chambers (Liao et al. 2013). All these approaches can be combined in a multi-step system as needed. The experimental examples presented in this review and their respective experimental parameters are compiled in Table 6.

**Table 6:** Parameters and results of described experiments

Experiment by	Geometry [µm]	Cell types	Freq [kHz]	Voltage [V_pp_]	σ_Medium_ [S m^-1^]	Flow [µL h^-1^]	Cell concentrations [ml^−1^]	Sep. efficiency
Han et al. (2011)	*W* = 30	All blood cell subtypes (separately)	100	5.8	σ_M_= 0.17	ѵ_S_ = 7 v_M_ = 70	Blood cells: medium 1:10	Size separation, each cell type separately
Han et al. (2008)	*W* = 100, *l* = 100	RBCs from WBCs	2 000	3	σ_M_ = 1.7	50	2.88 × 10^9^ *	A) separation efficiency of 93.6 % for RBCs and 76.9 % for WBCs B) 87.0 % for RBCs and 92.2 % for WBCs
Yakusawa et al. (2020)	*W* = 680, *h* = 50	RBCs and Monocytes (THP-1)	25	10 – 16, 30	σ_M_ = 0.051 PBS with 200 mM sucrose	18.36	RBC: 10^8^ TPH-1: 2 × 10^6^	RBCs: 91 % THP-1: 93 %
Piacentini et al. (2011)	*W* = 40, h = 40	PLTs from RBCs and WBCs	100	10	σ_M_ = 0.055 PBS, sucrose and 1 % of bovine serum; osmolarity = 0.3 Os ml^- 1^	*ѵ*_*S*_ = 0.77 ѵ_B_ = 4.81	1 – 2 × 10^8^	Purity (PLTs) = 98.8 %
Yang et al. (2000)	IDE, W = 50; *h* = 420, *w* = 2.5 × 10^4^, *l* = 3.88 × 10^5^	T- and B-Lymphocytes, monocytes, granulocytes	5 – 60	4	σ_M_ = 0.01 8,5 % sucrose, 0.3 % dextrose	1.2 × 10^4^	0.5 – 2 × 10^6^	87 % – 98 % (T-Lympocytes and granulocytes/monocytes)
Liao et al. (2013)	*H* = 30, w = 2 × 10^3^, l = 5 × 10^3^	Plasma/RBCs from whole blood	100 000	20	Whole blood	Capillary force	Hematocrit of 10 % – 50 %	67 % – 90 %
Pommer et al. (2008)	W = 1.75 × 10^3^	PLTs from whole blood	1 000	100	σ_W_ = 1.7 σ_B_ = 0.05 0.7:10 (whole blood:buffer)	150	8 × 10^7^	95 %

*assumed

**Fig. 4:**
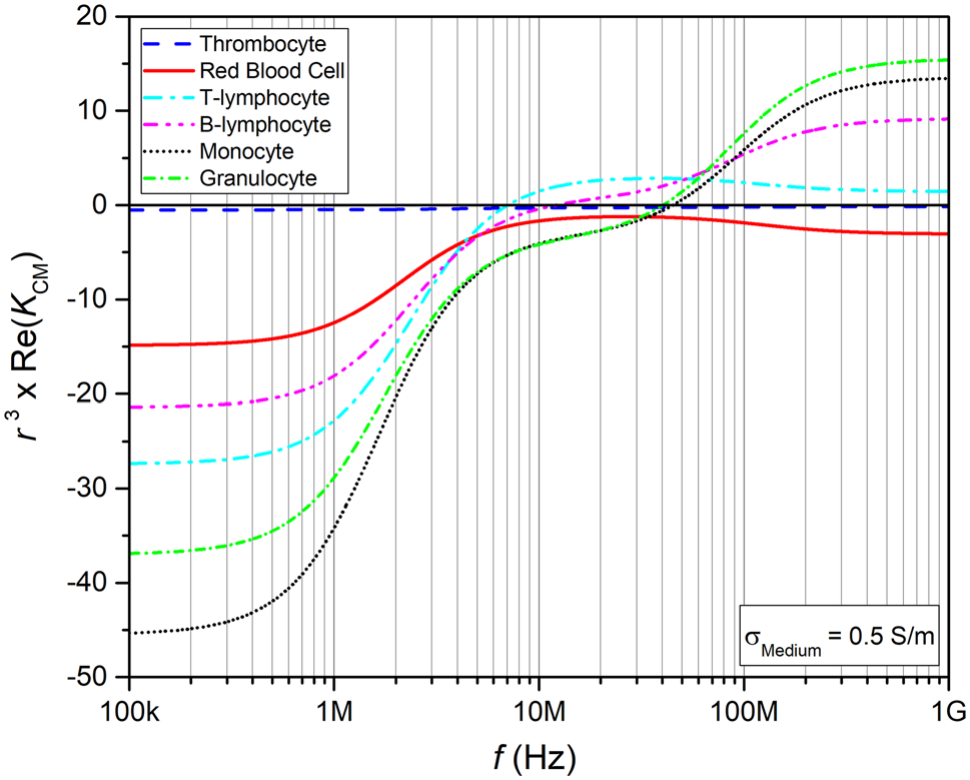
Cell size times Re(K_CM_) of blood cells in a low conductivity medium. Re(K_CM_) values calculated using the MyDEP software (Cottet et al. 2019), and multiplied by the cell size r^3^, the volume fraction is set to v = 0.45, the cells are each defined by a single shell model. The crossover frequency can be seen by the transition of negative to positive values

The frequency at which the devices are operated determines the magnitude of **F**_DEP_, because as mentioned above it depends on the Re(K_CM_). However, the **F**_DEP_ also scales with particle size r^3^. The combination of Re(K_CM_) and r^3^ is depicted in Fig. 4 (Cottet et al. 2019).

It can be seen from the figure, that although the Re(*K*_CM_) is identical for the blood cell subtypes at 100 kHz the DEP force ***F***_DEP_ as exerted on a single cell is not. In fact, at this frequency the difference and magnitude of ***F***_DEP_ takes its largest value.

### Inclined angle electrode geometry

In order to separate cells using negative dielectrophoresis, the geometry of the electrode beams can be set at an inclined angle to the flow profile. The cells are deflected along the electrodes into an adjacent laminar flow or towards a designated outlet.

**Fig. 5:**
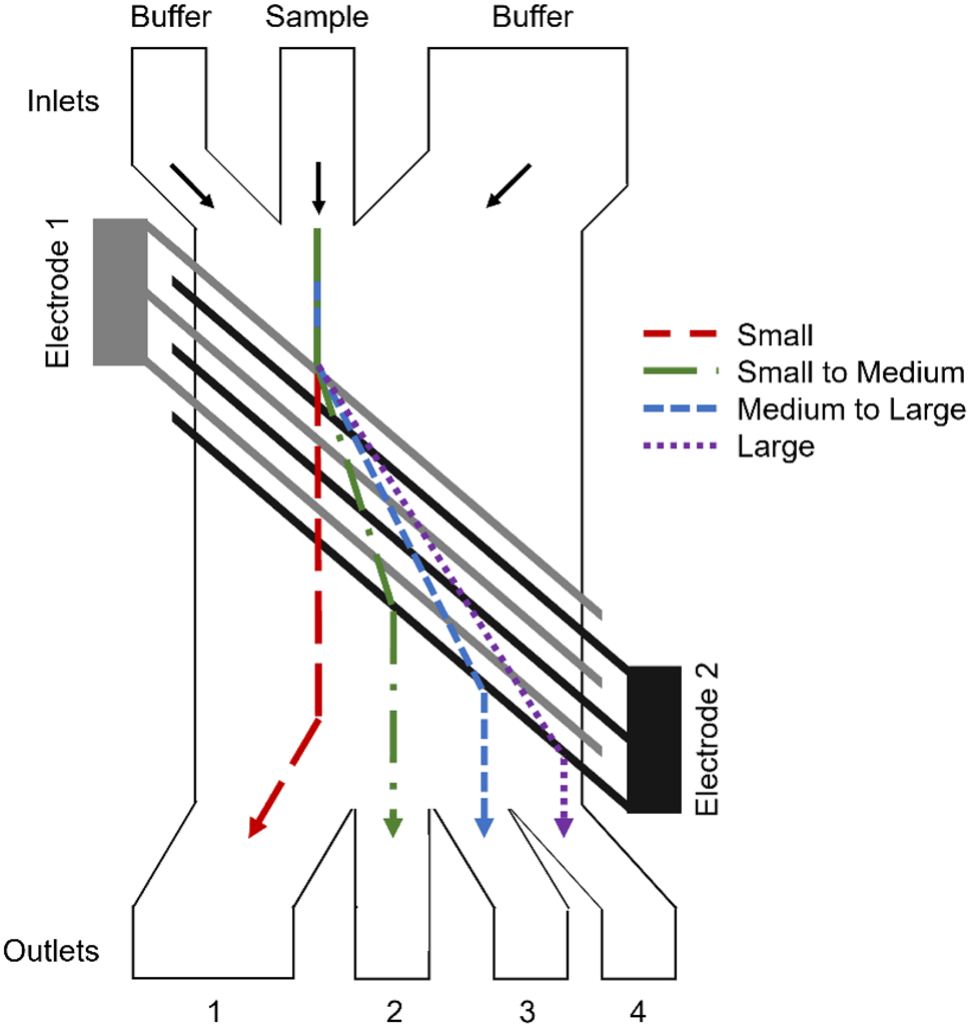
Device with an interdigitated electrode array that is orientated in the angle ϴ in direction of the flow. Cells of a greater size experience a stronger abbreviation and are therefore directed to outlets 2, 3 and 4. Smaller sized cells experience a lower lateral displacement and are therefore directed towards outlet 1 due to the fluid flow. Based on (Han et al. 2011)

This system is used in the lateral dielectrophoretic microseparator by Han et al. (2011). It has two buffer inlets located at both sides of the channel for a microfluidic manipulation of the cells trajectories. The focused cell stream is then exposed to an inhomogeneous electric field while passing over the inclined interdigitated electrode (IDE) structure. This allows the dislocation of the cells along the electrode beams into separate streams and subsequently into separate outlets, depending on their dielectric parameters (Fig. 5) (Han et al. 2011).

The IDE array is set at an angle of 45° to the flow and because it is operated at 100 kHz it leads to a size separation of blood cells.

This allows the efficient separation of platelets and red blood cells from monocytes and granulocytes, but not completely from B and T cells as their differences in cell size are too little (Han et al. 2011).

**Fig. 6:**
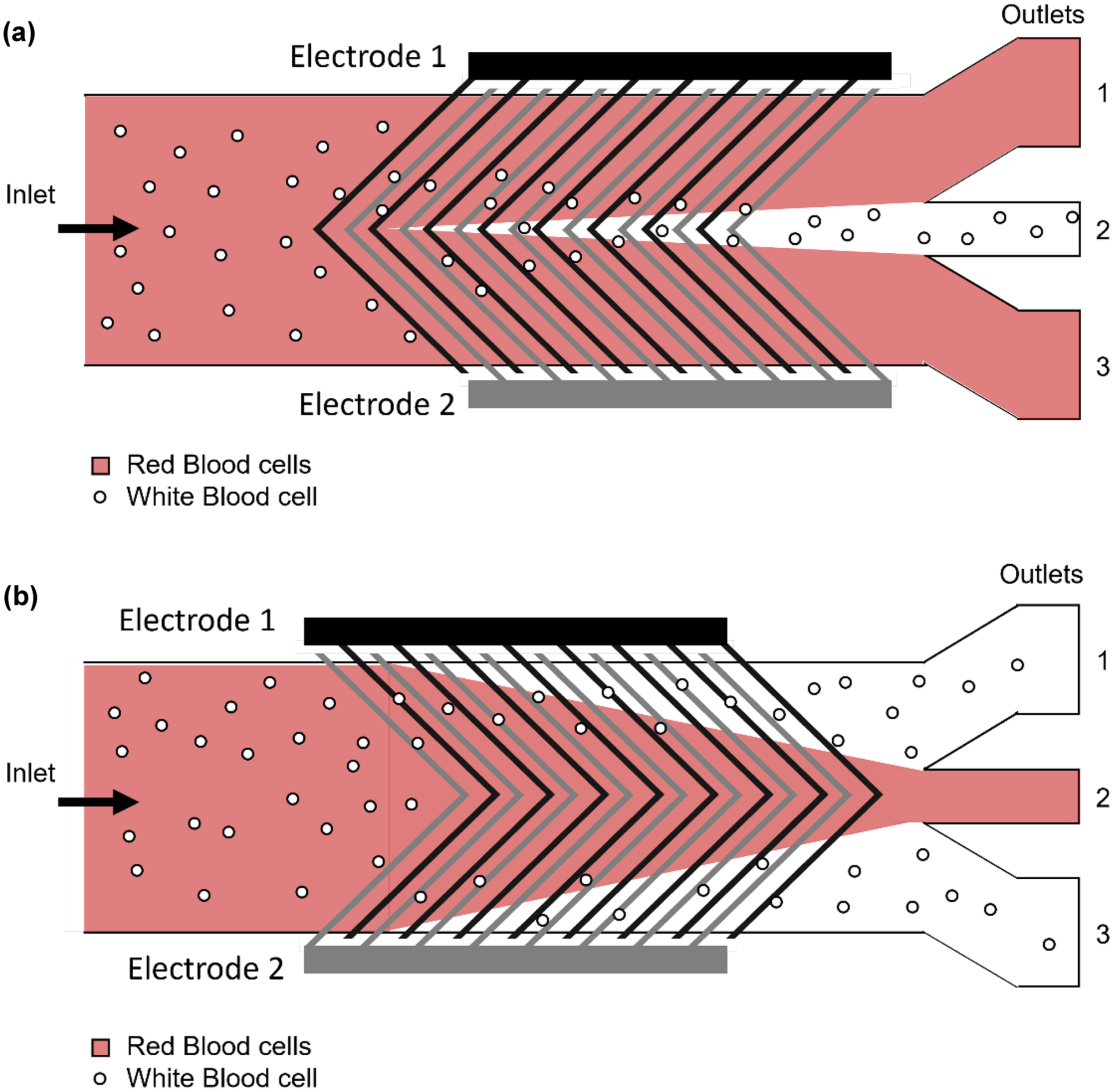
**(a)** Convergent mode in which RBCs (red) are directed towards outlet 2 and WBCs (orange) are directed to outlets 1 and 3. **(b)** Divergent mode in which RBCs are directed towards outlets 1 and 3 whereas WBCs are directed to outlet 2. Based on (Han and Frazier 2008)

Alternatively, the electrode beams can be designed in a herringbone formation as seen in the microfluidic DEP device by Han and Frazier (2008), see Fig. 6. The electrode.

geometry is described as either convergent (Fig. 6A) or divergent (Fig. 6B) with respect to the direction of the fluid flow. The device allows the separation of RBCs from WBCs. The RBCs experience a stronger deflection than the WBCs and are therefore directed along the electrodes towards either the top and bottom outlets see Fig. 6A or towards the middle outlet in Fig. 6B. An advantage of this setup is the ability to use highly conductive media which is closer to physiological conditions (Han and Frazier 2008).

The angled electrode geometry to separate RBCs and WBCs was used by Yasukawa et al. (2020) as well. They used a top bottom electrode geometry which allowed an increased channel height because of the wider special distribution of the inhomogeneous ***E*** field. When using this geometry, a singe electrode is already sufficient if the corresponding partner electrode on the other side of the channel has a opposite polarization.

**Fig. 7:**
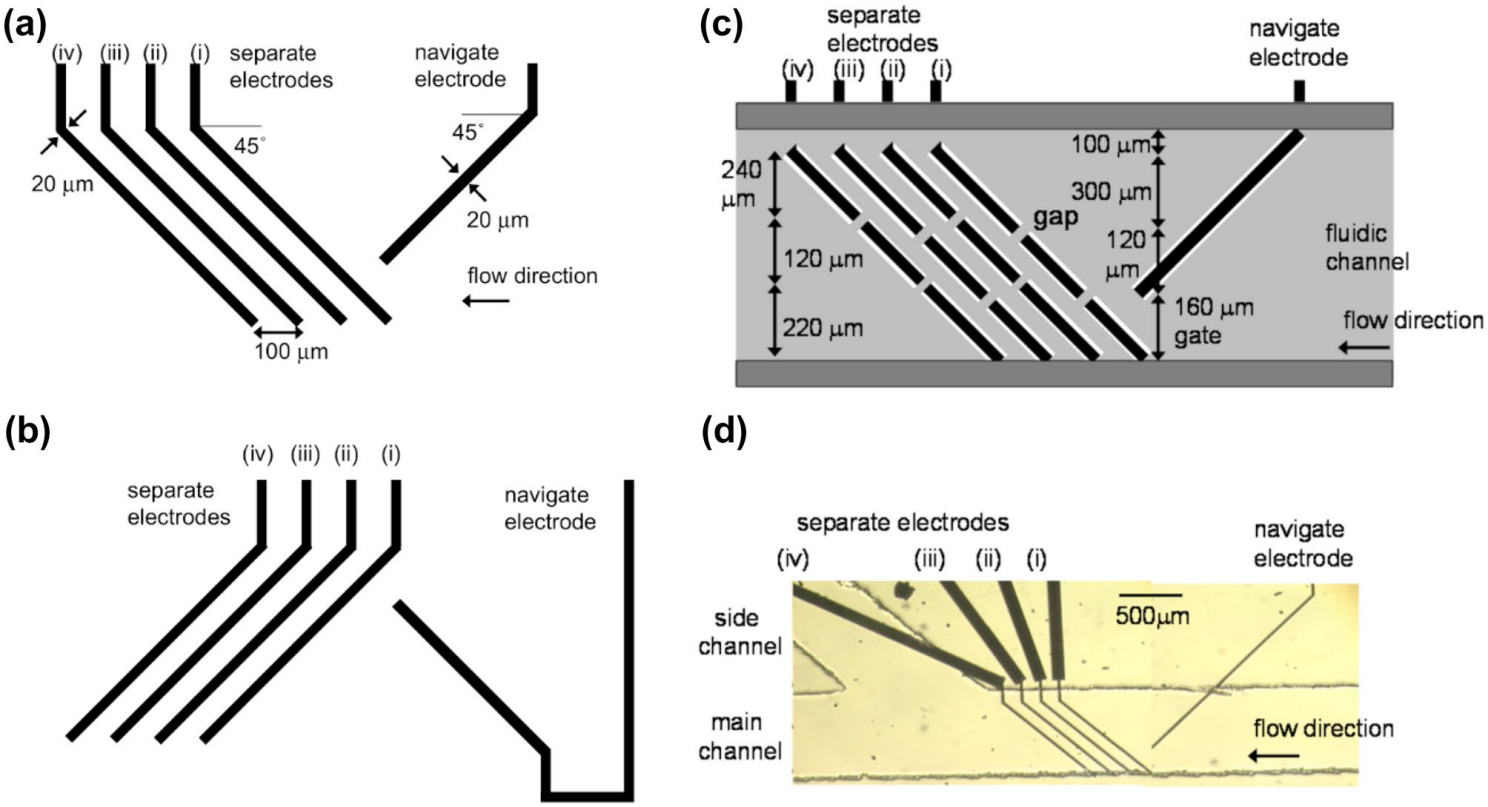
Electrode geometry of navigation and separation electrodes **(a)** Bottom and **(b)** Top electrode geometry **(c)** the insulating layer (light gray) and double-sided adhesive films (dark gray) on top of the bottom substrate **(d)** photograph of the bottom substrate and film for main and side channels. Reproduced from (Yasukawa et al. 2020) 2020, MDPI Publishing

The implemented device design is depicted in Fig. 7. Its dimensions are 680 × 50 μm and it includes two electrode types. One to navigate the cells into a single cell stream which is operated at 25 kHz and U = 30 V_pp_ and another electrode array in an 90° angle to the first electrode pair which separates the cells. Due to gaps in the electrode beams the separation is based on a size distribution of the cells. The separation electrodes are operated at an frequency of 25 kHz and a voltage of 10–16 V_pp_ (Yasukawa et al. 2020).

The blood cells are focused into a cell stream along the navigator electrodes and then separated due to the separation electrodes. They include gaps wide enough for RBCs to fit through. The monocytic leukemia cell line THP-1 act as a model for monocytes and are deflected along the electrodes into a side channel. The researchers achieved separation efficiencies of 91% RBCs by passage through the electrode gaps and 93% for the THP-1 cells, which arrived at the channel edge (Yasukawa et al. 2020). The high voltages at low frequency pose the danger of electroporation on the cells, however this issue was discussed in the publication and presumed to be of low risk. Additionally, the power dissipation of the ***E*** field to heat is limited due to the low conductivity medium, which allows the use of higher currents. However, it also requires pretreatment of the blood samples.

### Perpendicular electrode geometry

The electrode geometry can alternatively be set at an angle of 90° i.e., perpendicular to the flow direction. The cells are not deflected along the electrode beams but away from the electrode array. There are three subtypes of this system. The cells can be pushed away from so called liquid electrodes, which are set at the bottom of dead-end chambers on one or two sides of the microfluidic channel. These simulate 3D electrodes to a limited channel height. 3D electrodes would span across the channel side and can be fabricated using suspended conductive particles in a photoresist i.e., AgPDMS, which are silver particles suspended in PDMS (Jia et al. 2015; Lewpiriyawong et al. 2011), carbonised SU8 (Martinez-Duarte et al. 2010; Park and Madou 2005) or doped silicon (Iliescu et al. 2005) which are then structured using a mold or by developing the substrate. Alternatively, conducting 3D elements can be included in the design (Lewpiriyawong et al. 2010; Li et al. 2013b; Voldman et al. 2002; Wu et al. 2012). The third alternative is an interdigitated electrode array along the bottom of the microfluidic channel leading to various levitation heights of the cells.

#### Liquid electrodes

**Fig. 8:**
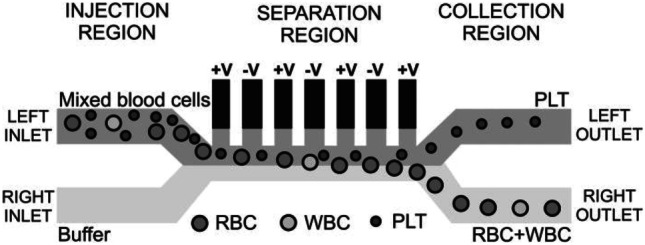
Cell separation of a mixture based on the cell size. The chip includes liquid electrodes (planar electrodes in dead-end chambers), the microfluidic channels are 40 µm high and 40 µm wide. The behavior of RBCs, WBCs and PLTs in the chip is depicted. Reproduced with permission (Piacentini et al. 2011) Copyright 2011, AIP Publishing

The term ‘liquid electrodes’ was coined by Demierre et al. (2007) describing electrodes in dead end chambers along the side of a microfluidic channel. The resulting ***E*** field which impacts the passing cells is inhomogeneous because of the isolator geometry which forces the current to travel along the main channel into the next niche, respectively. The strength of the ***E*** field diminishes with distance to the edge of the electrode array (Demierre et al. 2007). An adaption of this system to blood cells was published by Piacentini et al. (2011). It consists of an injection region with two inlets, a separation region and a collection region with two outlets, see Fig. 8 (Piacentini et al. 2011).

The cell mixture enters the device from the left inlet. The cells are then hydrodynamically focused by the buffer flow from the right inlet towards the left side of the microfluidic channel when entering the separation region. The buffer inlet has a more than six-fold higher velocity than the cell mixture. Piacentini states that these different velocities generate two separate laminar flows in the separation region, exhibiting small Reynold’s numbers of < 0.05 and facilitating cell separation. Additionally, the cell focus due to the second inlet leads to a similar starting place in the ***E*** field for all cells. The device is operated at 100 kHz, which leads to a size separation of the cells, see above. It is possible to separate PLTs from RBCs and WBCs with a purity of 98.8%. This is because after the microfluidic focusing of the cells, only RBCs and WBCs experience a significant negative DEP effect due to their larger cell size. They are deflected towards the right side of the separation region and into the right outlet. Because of the small cell size of the platelets the DEP force acting on them is negligible and they are directed by the force of the flow straight towards the left outlet (Piacentini et al. 2011).

#### Cell levitation

Cells can be repelled from an electrode array using levitation as well. It is an upward lateral displacement and can be generated by electrodes usually an IDE located at the bottom in a microfluidic channel. They create a negative DEP effect and a negative buoyancy force on the particles passing above them (Pethig 2017d).

The levitation can be calculated by

6 $${{h}}_{\mathbf{e}\mathbf{q}}={ }{{d}}_{0}+{ }{{d}}_{1}{ }\cdot ln\left(\frac{{{U}{\alpha }}_{DEP}({f})}{{\rho }_{{M}}-{\rho }_{{P}}}\right)$$

where *d*_0_ and *d*_1_ are constants that depend on the arrangement of electrodes and the permittivity of the medium (Wang et al. 2000). *U* represents the applied voltage and α the dielectrophoretic polarization factor, which depends on the applied frequency. The term ${\uprho }_{\mathrm{M}} - {\rho }_{\mathrm{P}}$ reveals that the levitation depends on the difference in density between medium and particle (Wang et al. 1998).

Based on the lateral displacement and the gravitation within the dielectrophoretic channel, different cells experience different cell trajectories within the flow of the DEP device (Yang et al. 2000). The resulting cell height determines the velocity of the cell in the channel due to the parabolic flow profile present in the microchannel (Khoshmanesh et al. 2011). This effect is described as field flow fractionation (Yang et al. 2000).

The velocity of the particle within the microfluidic channel is given by Eq. (7) (Gascoyne et al. 2002)

7 $${\nu }_{Cell}{ }=6{ }{\cdot \nu }_{mean}\cdot { }\frac{{{h}}_{\mathbf{e}\mathbf{q}}}{{H}}\cdot \left(1-{ }\frac{{{h}}_{\mathbf{e}\mathbf{q}}}{{H}}\right)$$

where *v*_mean_ describes the mean velocity within the channel and *H* is the height of the microchannel.

**Fig. 9:**
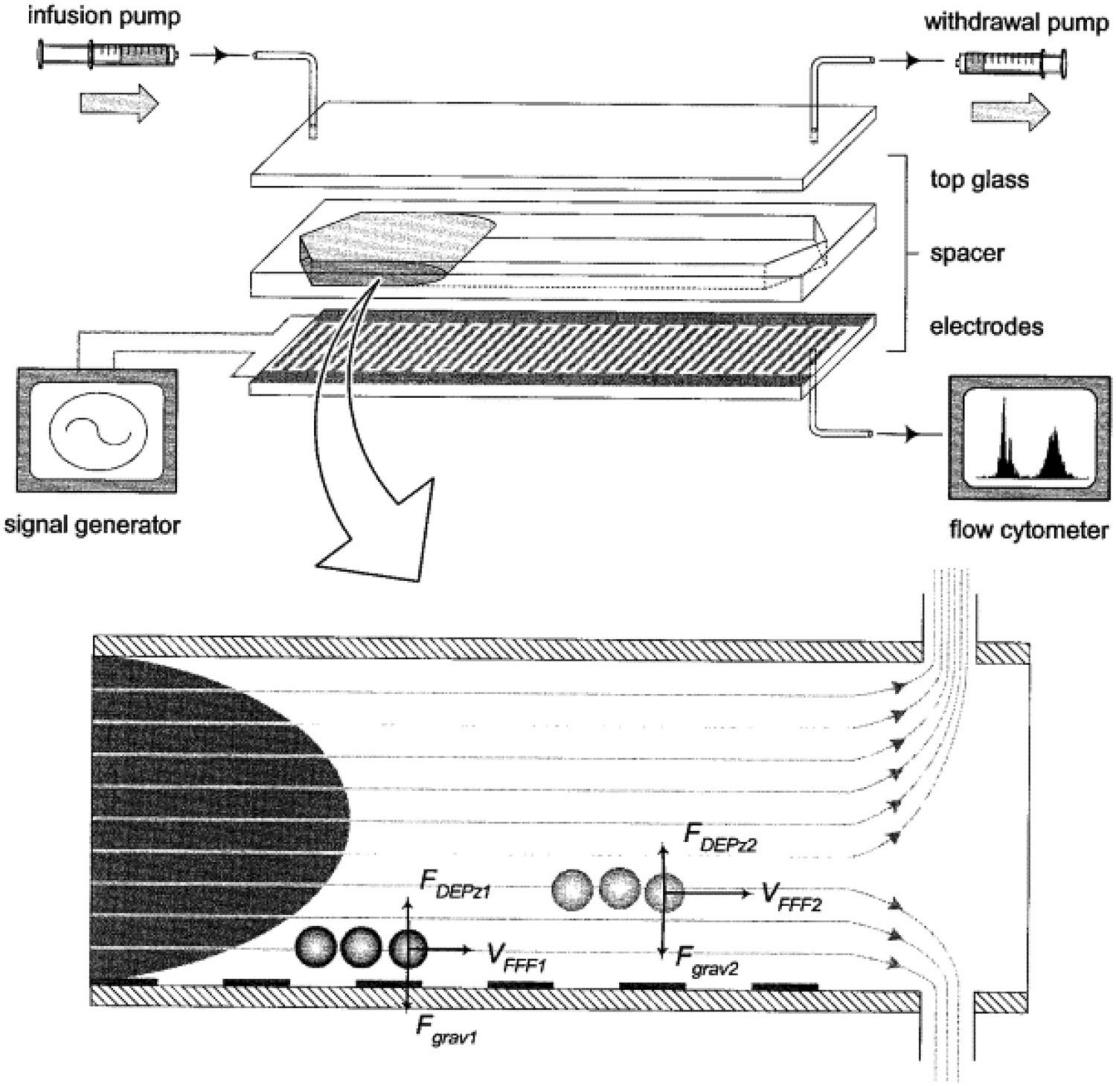
Displacement of particles within a DEP field. Experimental set up of a microfluidic chip with an interdigitated electrode array on the bottom and the forces acting on particles in the fluidic chamber. Particles with different dielectric parameters are levitated to different heights in the chamber and therefore subjected to different flow velocities. Reproduced with permission (Yang et al. 2000) Copyright 2000, Elsevier

Yang et al. have published a system using this approach (Yang et al. 2000). Their device consists of a separation region with an interdigitated electrode array, an inlet with an infusion pump, an outlet which is directly connected to the flow cytometer and a withdraw pump to produce a continuous flow, see Fig. 9 (Yang et al. 2000). The DEP field is generated by eight interdigitated electrodes with an element width and spacing of 50 µm that are located at the bottom of the microchannel with dimensions of *h* = 0.42, *w* = 25 and *l* = 388 mm. For separating the cell suspension, the applied swept-frequency of 5 – 60 kHz and an applied voltage of 4 V_pp_ was switched for the separation of each cell subclass.

Different cell velocities result in different elution times that can be used to separate cells in a similar approach to chromatography based on their lateral displacement and the sedimentation (Yang et al. 2000).

The relation between levitation and elution time can be shown in a force diagram (see Fig. 9). Both the levitation force and the sedimentation force are acting on the particle. The levitation force depends on the size and the density ρ of the particle (see Eq. (6)). The velocity shown in the flow profile in Fig. 9 depends on the height of the particle within the channel (Pethig 2017d).

With *L*, the length of the channel, the elution time τ of the specific cell type can be approximated, see Eq. (8) (Gascoyne et al. 2002)

8 $$\uptau ={ }\frac{{L}}{{\nu }_{Cell}}$$

The results of this study allowed the creation of a fractogram of the individual elution times as a function of frequency. This device has been able to separate monocytes or granulocytes from T- or B-Lymphocytes with a separation purity of 87 to 98% (Yang et al. 2000).

### Cell trapping

**Fig. 10:**
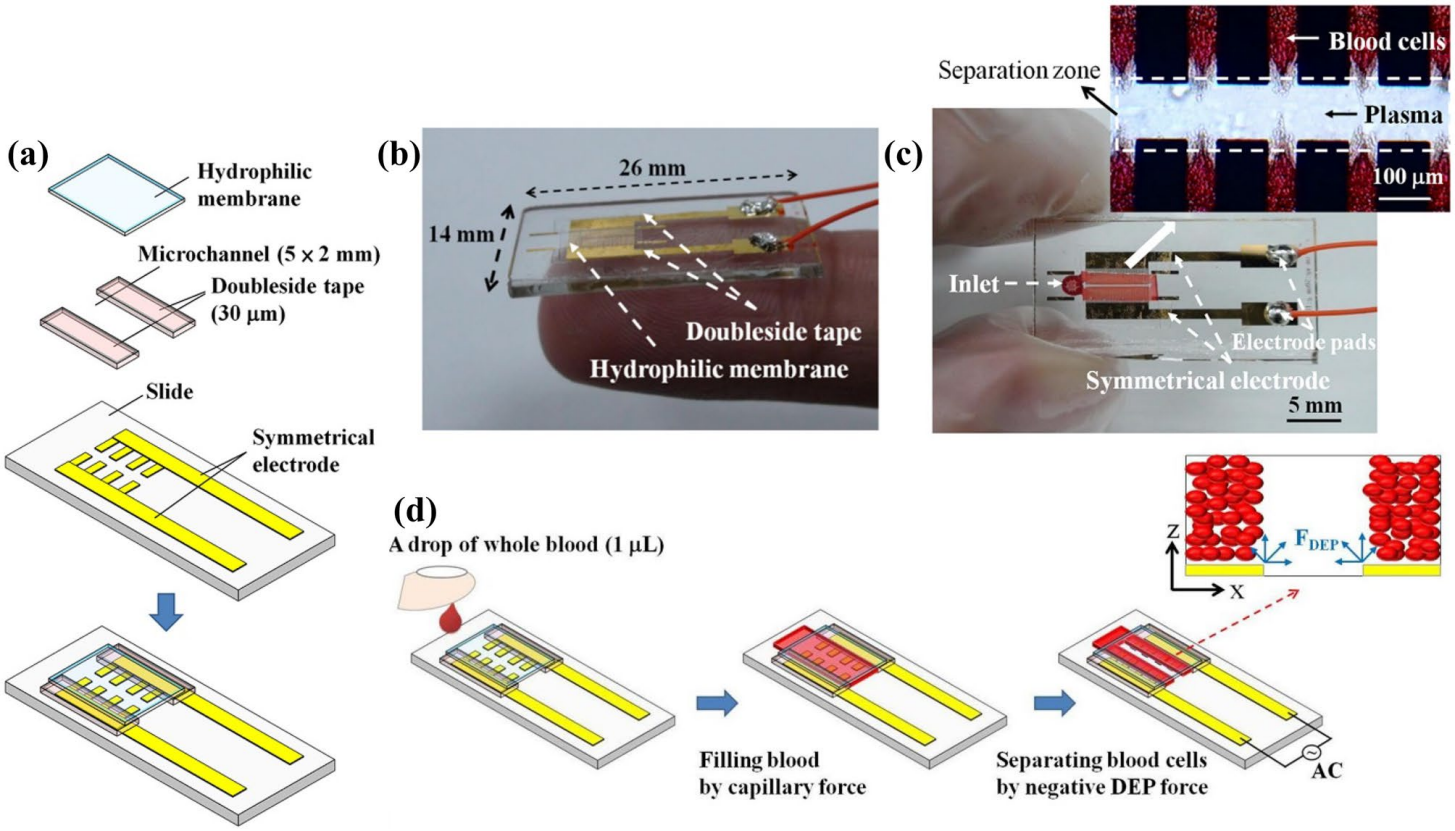
Experimental set-up of a capillary DEP device separating cells from the plasma. Reproduced with permission Copyright 2013, AIP Publishing

The DEP force can be adapted to trap cells for single cell analysis or to filter blood and separate cells from a heterogeneous suspension. The quadrupole is a prominent example of this approach and has been used in various experiments for various cell types (Burgarella et al. 2010; Pommer et al. 2008). Another approach using cell trapping was described by Liao et al. (2013). Their microcapillary DEP device with dimensions of *h* = 30 µm, *w* = 2 mm and *l* = 5 mm as shown in Fig. 10 is capable of the separation of blood cells from blood plasma by applying a voltage of 20 V_pp_ and a frequency of 100 000 kHz.

This set-up represents the only example, where instead of microfluidic pumps capillary forces were exploited to drag the whole blood sample of around 1 µL into the device and the cells.

were collected at the electrode gaps by an attractive dielectrophoretic force, positive DEP. This device has no need for pumps or sample preparation prior to cell separation but is not capable of separating different types of cells. Liao et al. also showed with this experiment the importance of the hematocrit, which influences the separation efficiency. They found, that the lower the hematocrit of the blood sample is, the faster the sample is reaching a higher separation efficiency (Liao et al. 2013). Their approach is useful for removing cells from a solution like blood plasma, which is an improved approach compared to centrifugation.

### Combination of different techniques

**Fig. 11:**
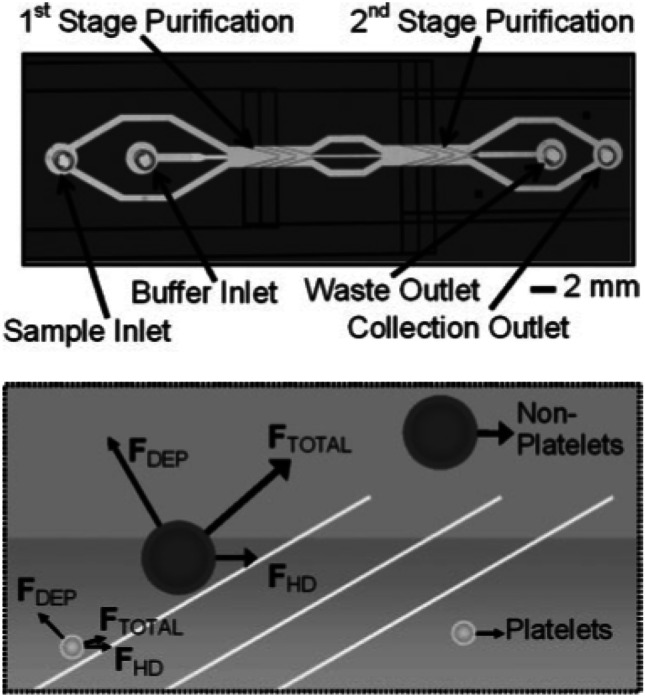
Architecture of the two-staged dielectrophoretic device and its separation mechanism. Cells that are non-platelets are experiencing a negative DEP effect and are deflected by the electrodes into the buffer stream which directs them into the waste outlet. Platelets are only influenced in a negligible way due to their small cell size and are not reflected by the electrodes and directed into the collection outlet (Pommer et al. 2008) Copyright 2008, Wiley

Electrode configurations can be added or combined in order to make use of more complex separation schemes. One example, where this approach was implemented is the two-staged microfluidic DEP device by Pommer et al. (2008). It consists of an injection region with two inlets, two purification stages for the separation of cells, and two outlets (Fig. 11) (Pommer et al. 2008).

An additional purification stage was added to enhance the purity of the separated thrombocytes in the collection outlet. Larger cells experience a greater negative DEP effect and are deflected into the buffer stream and therefore directed into the waste outlet. Due to the small cell size of platelets, they are only reflected by the electrodes in a negligible way and thus are directed in the collection outlet. This allows the separation of platelets from other blood cells. For the detection and identification of the thrombocytes an integrated optical microscope was used. The separation of cells was conducted with two different buffers and their separation efficiencies were compared. The lower conductivity buffer allowed the usage of a higher voltage of 100 V_pp_ without electrolysis of the cells (Pommer et al. 2008).

## Conclusions and perspectives

Dielectrophoresis has been used in a number of experiments to separate blood cells in recent years. The method mainly exploits the different dielectric constants ℇ and conductivities σ of thrombocytes and red and white blood cells. Due to the short range of the DEP force, the electrodes have to be integrated into microfluidic devices, so that the volume throughput has not yet reached the medically relevant range of µL min^−1^. The throughput could be increased by using 3D electrodes which could possibly reduce the processing time, but at the cost of an increased fabrication complexity (Techaumnat et al. 2020; Zeinali et al. 2015). Another option to increase the Lab-on-Chip throughput might be the parallelization of several microchannels, however this would again require a more costly fabrication. Most of the devices shown in this review focused on an interdigitated electrode array and used frequencies between 20 kHz to 2 MHz. The use of lower frequencies allows the separation based on different sizes of blood cells due to identical Clausius Mossotti factors at frequencies below 100 kHz in most buffers and blood plasma.

However, the use of DEP-based separation holds the promise of simplified sample preparation. Successful experiments have been demonstrated, in which labeling with specific antibodies, washing and blocking of the antibodies could be fully dispensed with. Therefore, the use of these DEP devices is less time consuming, and results of subsequent analyses might be more reliable as the cells are in a more natural state.

The studies conducted have shown that the separation efficiency of DEP devices depends on the used buffer, the electrode and microfluidic channel geometry, the total experimental set-up of the device and the applied voltages and frequencies. It is important to consider both Joule heating as well as electroporation and -lysis when designing and defining the desired device, which depend on the maximum applied field in relation to the conductivity of the medium, the size and dielectric parameters of cells and the applied frequency. So far, the miniaturization of DEP-based devices to make them appropriate for PoCT diagnostics is also hindered by large form factors of both microfluidic pumps and electrical generators (Jung et al. 2015).

The complexity of the separation system to be set up increases the more the dielectric parameters of the cells to be separated resemble each other and the more cell types are to be separated. Various devices are already capable of separating different types of cells, but none of these devices are yet capable of separating all types of blood cells. For instance, no complete separation of leukocyte subtypes by microfluidic DEP devices has been achieved to date, especially the separation of T and B cells remains challenging. Additionally, none of the discussed devices can perform the separation of cells without additional larger scale laboratory devices yet. To improve separation, various frequencies could be applied as well as the use of additional stages, either by combining approaches or repeating geometry elements (Khoshmanesh et al. 2011; Yang et al. 2000). Last, but not least, a DEP-based blood cell separator would require additional stages for counting individual cell fractions, which would be needed for diagnosing possible diseases the patients are suffering from.

In the future DEP-based LoC systems will be designed to work on a small scale. That means, that the devices are transportable and are therefore not restricted to laboratories only, but can be used in every environment, which enables Point-of-Care-Testing (PoCT) (Jung et al. 2015). Those devices could facilitate fast and reliable diagnosis of a diseases. Microfluidic DEP devices have already proven to be able to separate circulating cancer cells from blood cells (Aghaamoo et al. 2019; Becker et al. 1995), as well as Malaria-infected erythrocytes from healthy ones (Gascoyne et al. 2004, 2002). This might be of special relevance for e.g., the diagnosis of leukemia, improving result generation and the cost of a blood test could potentially be minimized by DEP LoCs instead of using hemacytometers. Moreover, PoCT is especially important in emergency scenarios and in geographic regions with a low level of medical infrastructure.

Further applications will arise from new methods under development in materials science for fabricating microfluidic systems with integrated metal electrodes that will become available for low-cost fabrication. In addition, DEP will be able to develop a major advantage for establishing itself as a widely used method for cell separation when combined with microelectronics. This is due to its electrical nature, enabling the method to be integrated with microchip-based techniques for control and data analysis in extremely miniaturized lab-on-chip systems (Babay et al. 2020; Birkholz et al. 2016, 2010; Lacroix et al. 2019). It can thus be expected that the merging with microelectronics will pave the way for an increased use of DEP-based blood cell separators in medical research and patient care.
